# PATTERNS AND THEMES OF OLDER ADULTS’ COPING ACROSS TWO YEARS OF THE COVID-19 PANDEMIC

**DOI:** 10.1093/geroni/igad104.0242

**Published:** 2023-12-21

**Authors:** Heather Fuller, Andrea Huseth-Zosel, Emily Kinkade, Bryce Van Vleet, Melisa Hajdar

**Affiliations:** North Dakota State University, Fargo, North Dakota, United States; North Dakota State University, Fargo, North Dakota, United States; North Dakota State University, Fargo, North Dakota, United States; North Dakota State University, Fargo, North Dakota, United States; North Dakota State University, Fargo, North Dakota, United States

## Abstract

Throughout the COVID-19 pandemic, older adults developed coping strategies to adapt to the necessary social distancing precautions intended to protect from severe illness. However, over time, especially as vaccines became available, their need and ability to adapt and cope shifted. This longitudinal, mixed-methods study investigates whether older adults’ perceptions of coping changed across the first two years of the pandemic and how older adults described their experiences with coping at different points during this time. Between April 2020 and June 2022, five waves of interviews were conducted with 76 Midwestern older adults between the ages of 70 and 97. At each timepoint, participants rated their level of perceived coping, quality of life, and stress. They also answered several open-ended questions about their current daily life and experiences during the pandemic. Repeated-measures ANOVAs indicated participants’ perceived coping and quality of life significantly increased between the first and final interview. Thematic coding of interview transcripts identified four themes of coping across two years of the pandemic: 1) staying occupied, 2) seeking social support, 3) cultivating an adaptive mindset, and 4) future planning. Theme meaning shifted once vaccines were available, as participants adapted to ‘a new normal’ lifestyle, gained realistic perspectives, and appreciated their own resilience. Findings suggest older adults had nuanced and shifting coping experiences throughout the initial two years of the pandemic, but overall coped by drawing on life experiences. Our discussion will highlight variability in older adults’ coping over time and directions for future study and practice.

